# Use and Abuse of Poultices

**Published:** 1866-08

**Authors:** 


USE AND ABUSE OF POULTICES.—The *British Medical Journal* quotes some excellent remarks of Dr. Richardson from his lectures delivered at the College of Physicians on the use and abuse of poultices.

The application of moist heat in the form of poultice to suppurating parts, requires, he thinks, remodeling, in order that it may be placed on a true scientific basis. The common recommendation, "you must put on a poultice," is too often an easy way of doing something, about which we were not quite sure, and concerning which it were too much trouble to think long. Mischief is very often done by a poultice, which might well be avoided.

When a part is disposed to suppurate, the first step in the series of changes is an increased flow of blood through the capillary surface, followed by obstruction, and thereupon by an excess of sensible heat derived from the friction that is set up. Then follows transudation of liquor sanguinis into the connective tissue, and its transformation, under the influence of heat, into what is called purulent fluid. When to the part in this state we apply moist heat, we quicken suppuration, mainly by upholding the temperature: at the same time we secure the transference of water from the moist surface into the fluids of the inflamed part, by which tension of tissue is produced, and in the end yielding of tissue at the weakest point.

When the suppurating surface is circumscribed, the rapid induction of the process may be attended with little injury; but when the surface is large, and when the exuded fluid is thrown into loose structures where it can burrow readily, the practice cannot be good to the extent of the mischief. Hence in the treatment of carbuncle and plegmonous erysipelas, it cannot be sound practice in the early stage to apply moist heat. Experience as well as principle warrants this conclusion. In cases of carbuncle especially, Dr. Richardson has of late avoided the application of moist heat in the early stages with good results.

But when in the course of local disease, suppuration is actively established and is naturally circumscribed; when the increased temperature of the part has fallen to or below the natural temperature—then the value of moist heat comes on with full force. Then the tension which is exerted determines the escape of fluid at the weakest point of the surrounding tissue, and when the fluid escapes, or is liberated by the knife, the escape for a long period is aided by application of moist heat.

The continued application of moist heat for a long time after the escape of purulent fluid is again indifferent practice. It sustains discharge, it sets up unhealthy decomposition of fluids; it produces a thickened, soddened condition of skin, most favorable to the production of sinus; and it retards recovery. When a surface is freely open, and suppurating, dry and not moist heat is the remedy. We are in want in these cases of a simple invention; we require something which we can apply as readily as a poultice, which shall keep up the temperature of the part, and at the same time take up moisture, and gently desiccate, without injuring the tissues.

